# Soil Bacterial Communities from Three Agricultural Production Systems in Rural Landscapes of Palmira, Colombia

**DOI:** 10.3390/biology12050701

**Published:** 2023-05-11

**Authors:** Paula Andrea Rugeles-Silva, Jairo Andrés Londoño, Marina Sánchez de Prager, Jaime Eduardo Muñoz Flórez, Diana López-Álvarez

**Affiliations:** 1 Departamento de Ciencias Biológicas, Facultad de Ciencias Agropecuarias, Universidad Nacional de Colombia, Palmira 763533, Colombia; 2 Sección de Identidades Digitales, Universidad Nacional de Colombia, Palmira 763533, Colombia

**Keywords:** metabarcoding, agrosystems, management, soil

## Abstract

**Simple Summary:**

Three production systems with different management strategies were evaluated for soil bacterial diversity. Soil samples were taken from agroecological, organic, and conventional agricultural production systems, and physicochemical and metabarcoding analyses were carried out to evaluate if bacterial diversity was directly influenced by the management type. Differences in soil bacterial communities were not found to depend directly on the management, likely due to the non-aggressive practices used in all three systems. Rather, the differences can be attributed mainly to the use of different fertilizers rich in nitrogen and phosphorus and the use of pesticides.

**Abstract:**

Soils play important roles in the proper functioning of agroecosystems. Using molecular characterization methods such as metabarcoding, soils from eight farms (57 samples) belonging to three production system types—agroecological (two farms with twenty-two sampling points), organic (three farms with twenty-one sampling points), and conventional (three farms with fourteen sampling points)—were compared from the rural villages of El Arenillo and El Mesón in Palmira, Colombia. Amplification and sequencing of the hypervariable V4 region of the 16S rRNA gene was performed using next-generation sequencing (Illumina MiSeq) to estimate the bacterial composition and the alpha and beta diversity present. Across all soil samples, we found 2 domains (Archaea and Bacteria), 56 phylum, 190 classes, 386 orders, 632 families, and 1101 genera to be present. The most abundant phyla in the three systems were Proteobacteria, (agroecological 28%, organic 30%, and conventional 27%), Acidobacteria (agroecological 22%, organic 21%, and conventional 24%), and Verrucomicrobia (agroecological 10%, organic 6%, and conventional 13%). We found 41 nitrogen-fixing and phosphate-dissolving genera which promote growth and pathogens. Alpha and beta diversity indices were very similar across the three agricultural production systems, as reflected by shared amplicon sequence variants (ASVs) among them, likely due to the proximity of the sampling sites and recent management changes.

## 1. Introduction

Soil microbial diversity is considered a valuable source of microorganisms that can be beneficial to humans; however, to take advantage of this resource, it is necessary to comprehensively explore all microorganisms present in the soil. Identifying and characterizing microorganisms that belong to general groups can provide information about their roles and functions in their natural environments, which can help to formulate hypotheses about metabolic interactions between microorganisms in the natural environment [1].

Traditional farmers have developed complex productive systems that are characterized by a great biological and genetic diversity and adapted to the specific conditions of each system [2]. However, modern agriculture does not recognize the multifunctionality of these small systems that contribute to the protection of biological diversity, culture, and the local economy [3,4].

Bacteria perform various functions critical to soil and plant health, such as conversion and uptake of plant-available nutrients, stimulation of plant growth and resistance, and protection against plant pathogens. In addition, secretions from soil bacteria contribute to the formation of microaggregates, which improve soil structure, gas exchange, infiltration, and water retention. The diversity and composition of bacterial communities can provide valuable information about the health and functioning of associated environments, including agricultural systems and practices. It has been suggested that investigating soil bacterial communities may be a way to assess the condition of the soil and the productivity of the corresponding ecosystems, particularly in production systems where soil and plant health are closely related to productivity [5,6,7].

Changes in land use and the use of pesticides, tillage, and fertilizers have direct and important effects on soil microbiomes, including changes in chemical and physical properties, that directly affect soil biodiversity [8,9]. Metabarcoding is a technique that makes it possible to identify, from the isolation of DNA from soil samples, the untapped gene pool of microbial communities in the soil. Using this approach, alterations in soil microbial communities can be assessed according to the use of agricultural inputs [8]. The description of the microbiota associated with agricultural products is important for understanding the behavior of crops from the perspectives of phytopathology, horticultural sustainability, and food security [10].

Different agricultural practices can have varying impacts on the physicochemical properties of soil and vegetation, which in turn can affect biodiversity and ecological processes such as nutrient cycling and gas exchange. Although it is known that agricultural practices affect microbial communities, it is still not fully understood how specific production systems influence the structure of soil bacterial communities and what degree of variation exists between and within these communities. To understand these variations, further research across different settings is required [11,12,13,14].

The aim of this study was to characterize the microbial community of soils from three different production systems: (a) agroecological systems that accumulate organic matter, refrain from using agrochemicals, and practice polycultures; (b) organic systems that incorporate moderate usage of agrochemicals, monocultures, and bare soils; and (c) conventional systems characterized by the application of agrochemicals and monocultures [15]. These soils were compared to understand how the soil bacterial diversity is related to the different production systems. We hypothesize that agroecological systems have the most preserved soil microbial communities due to the exclusion of agrochemicals in their use.

## 2. Materials and Methods

### 2.1. Study Area and Sampling Sites

The study area is located in the Valle del Cauca department, in the municipality of Palmira, Colombia (Table 1). A total of eight farms were sampled that represent three different agricultural production systems. Two farms were sampled that are characteristic of agroecological cultural management, defined as systems with an accumulation of organic matter, no use of chemical products, polycultures, and family participation [11]. Three sampled farms represent organic agricultural systems, defined as those that have reduced their usage of synthetic fertilizers and pesticides, use a combination of traditional and modern technologies, and have weeds present [11]. Finally, three sampled farms are characterized as conventional systems, which include pest and disease control with agrochemicals, bare soil, weed control, and chemical fertilization [11].

### 2.2. Soil Samples

At each farm, sampling was carried out considering the associated crop, the total area of the land, and the slope, resulting in a total of 57 points selected (Table 1). Sampling was performed during the dry season (January 2016). A total of 1 kg of soil was collected from each point for physicochemical tests, while five sub-samples of 5 g each were taken for molecular analyses. Considering that several researchers suggest that soils harbor a greater quantity and diversity of microorganisms at depths of 0–15 and 15–30 cm [16,17,18], samples were taken at a depth of 30 cm. Sediment samples were stored at −80 °C until they were processed. Abiotic analyses were performed to measure physicochemical properties, including pH, organic carbon (OC), organic matter (OM), nitrogen (N), calcium (Ca), magnesium (Mg), potassium (K), sodium (Na), cation exchange capacity (CEC), phosphorus (P), copper (Cu), zinc (Zn), manganese (Mn), iron (Fe), boron (B), bulk density (BD), real density (RD), total porosity, macroporosity, microporosity, aggregate stability index (ASI), and available water (AW). These analyses were carried out in the Laboratory of Soil Chemistry and Physics at the Universidad Nacional de Colombia, Sede Palmira. For the biotic characterization of soil samples, a metabarcoding study was performed, sequencing the variable V4 region of the 16S rRNA gene.

### 2.3. DNA Extraction, Amplification, and Sequencing

Total DNA was isolated from 0.25 g of sediment for each sample using a DNeasy Powersoil Kit (Qiagen, Hilden, Germany), following the manufacturer’s instructions. The DNA quantity and quality were evaluated by spectrophotometry using a ColibriTM (Titertek Berthold, Neulingen, Germany) and 0.8% agarose gel electrophoresis using a standard protocol to evaluate sample degradation. A total of 57 barcoding libraries were constructed for the variable V4 region of the 16S rRNA gene with primers 515F (GTGYCAGCMGCCGCGGTAA) and 806R (GGACTACNVGGGTWTCTAAT) [12]. A 28-cycle PCR was performed using a HotStarTaq Plus Master Mix Kit (Qiagen, Hilden, Germany) under the following conditions: 94 °C for 3 min, followed by 28 cycles of 94 °C for 30 s, 53 °C for 40 s, and 72 °C for 1 min, after which a final elongation step at 72 °C for 5 min was performed. Library sequencing was performed at MR DNA (www.mrdnalab.com, accessed on 3 March 2017, Shallowater, TX, USA) using the Illumina MiSeq platform to obtain ~170,000 paired-end reads of 250 base pairs per sample. The Phred quality for the raw data was between Q32 and Q40 with an accuracy greater than 99.99%. The 16S rRNA gene sequences obtained in this study have been deposited in the European Nucleotide Archive (ENA) under project number PRJEB60414 and accession numbers ERS14885629 to ERS14885685 (Table S1).

### 2.4. Bioinformatics Analyses

Taxonomic analyses were performed using QIIME 2 release 2017.12 [19,20]. Raw sequence quality control was run with the DEMUX plugin, and reads were trimmed to a length of 250 base pairs using the DADA2 plugin [21]. Chimeric reads were discarded using the consensus method and amplicon sequence variants (ASVs) were determined and assigned to clusters using a QIIME feature that uses a naive Bayes classifier to map each sequence to taxonomy. The classifier was trained on QIIME-compatible Greengenes v13_8, refs. [22,23], with 99% similarity.

Statistical analyses were carried out in R v4.0.2 using the packages for principal coordinate analysis (PCoA), qiime2R v.0.99.23, phyloseq v1.30.0, and DESeq2 of Bioconductor. The resulting distance matrices were used for these calculations to compress the dimensionality into two dimensions, allowing visualization of the relationships between samples.

We calculated three alpha diversity indexes. The first is the Shannon–Weaver equity index, which measures the average degree of uncertainty in predicting to which species an individual chosen at random from a sample belongs.

$$H^{\prime }=-\sum \;pi-ln\;pi$$

where *pi* = proportional abundance of species *i*, based on the number of individuals of species *i* divided by the total number of individuals in the sample [24].

Simpson’s dominance index shows the probability that two individuals taken at random pertain to the same species and is strongly influenced by the dominant species.

$$\lambda =\sum \;pi^{2}$$

where *pi* = proportional abundance of species *i*, (i.e., the number of individuals of species *i* divided by the total number of individuals in the sample). A value of 0 indicates infinite diversity, while a value of 1 indicates no diversity [25].

Additionally, the Chao1 richness index estimates the number of expected species considering the relationship between the number of species per one or two individuals [26].

On the other hand, estimates of beta diversity were calculated within QIIME2 via q2-diversity using the Bray–Curtis distance between samples. A principal component analysis (PCA) was conducted using the FactorMiner R package [27,28] to simultaneously analyze the 17 physicochemical variables and 27 bacteria.

Finally, the richness was determined considering the number of species per sampling site in a Venn diagram using the jvenn interactive program to identify both the unique species and shared species per system [29].

## 3. Results

### 3.1. Physicochemical Soil Analyses

The average values of the soil properties in each production system are presented in Table 2. In general, it is observed that the soil of the three systems had similar characteristics, with a slightly acidic pH ranging between 6.08 and 6.60. We observed that organic matter, nitrogen, organic carbon, Mg, and CEC are higher in agroecological systems, while Ca, K, and Na are higher in conventional systems. Potassium was found in similar amounts in agroecological and organic systems, but in conventional systems, this mineral was found in excessive levels. Regarding the microelements Fe, Cu, and Mn, high values were observed in the organic systems.

With respect to the physical properties of the soil, the values obtained for bulk density, real density, and microporosity were similar across the three production systems, while the total porosity was slightly higher in the organic system than in the other two systems (Table 2). On the other hand, microporosity exhibited lower values in the conventional system than in the agroecological and organic systems, but higher values in the usable sheet of water. Regarding the stability index, the conventional and organic systems presented higher values of 0.45 and 0.44 in comparison to that of the agroecological system, 0.19. The reported values of the stability index were less than 1 in all cases, indicating that the sampled soils are more prone to erosion either by mass movements or by dragging fine particles [30].

The bulk density (BD) values obtained (i.e., space occupied by the pores of the soil sample) are negatively correlated to the organic matter (OM) present, i.e., a higher OM causes a lower BD [30]. Micropores predominate in all three systems, indicating that the soils have good moisture retention and moderate drainage. When analyzing the physical and chemical variables according to the USDA, the sampled soils are classified within the Inceptisols soil order, which is characteristic of humid climates under an udic moisture regime and contain a high base saturation [31]. The texture of the soils is of the clay loam type with a good water retention capacity, since soils with high clay content retain twice as much water, a condition that tends to favor the cation exchange capacity.

### 3.2. Bacterial Composition Analysis

A total of 22,333,710 sequences were obtained across samples, which were assigned to 22,251 ASVs with a confidence rate between 70 and 100%, except for 25 unknown assignments. A total of 2 domains (Archaea and Bacteria), 44 phyla, 122 classes, 194 orders, 212 families, and 265 genera were identified.

**Figure 1:**
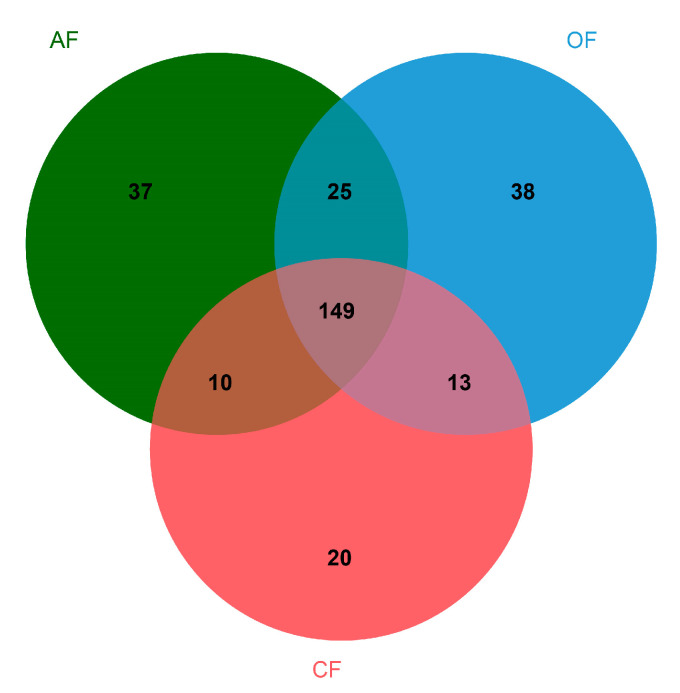
Taxonomic distribution comparison of genera among the three agricultural production systems.

At the genus level, 149 were found to be shared among the three production systems, while 37 were unique to AF, 38 to OF, and 20 to CF (Table S2 and Figure 1).

**Figure 2:**
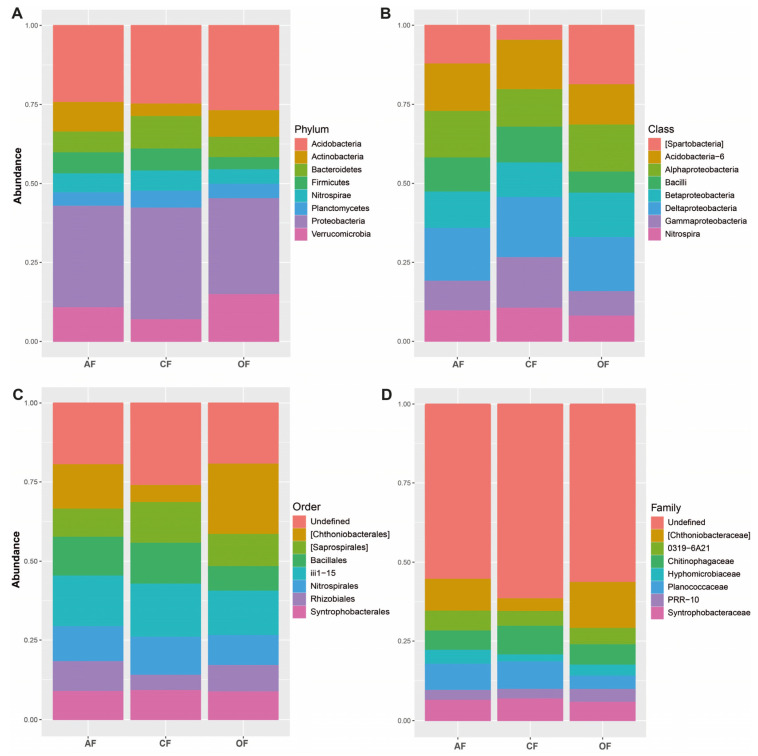
Relative abundance of bacteria in the three agricultural production systems, classified by (**A**) phylum, (**B**) class, (**C**) order, and (**D**) family.

Proteobacteria (AF 28%, OF 30%, and CF 27%), Acidobacteria, (AF 22%, OF 21%, and CF 24%), and Verrucomicrobia (AF 10%, OF 6%, and CF 13%) were the most dominant phyla of the bacterial communities present in the soils of AF and OF systems; CF shared similar abundances of proteobacteria and Acidobacteria with the other two systems, but differed by presenting a higher relative abundance of Bacterioidetes (Figure 2).

**Figure 3:**
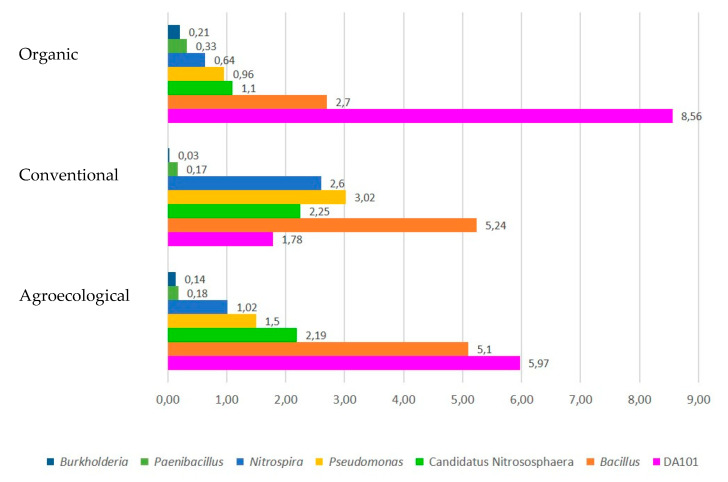
The most abundant genera found within the soils of the three production systems.

The five most abundant genera in the three systems are observed in Figure 3. DA101 (Candidatus Udaeobacter copiosus), a genus related to grassland species [32], was found with a greater abundance in OF systems, perhaps because they have the greatest presence of grasses. *Bacillus* and *Pseudomonas* were also found in greater abundance in CF and AF systems. These genera are free-living bacteria related to plant growth [33,34]. On the other hand, Candidatus Nitrosphaera was identified to occur more frequently in AF systems, being an Archaea related to the oxidation of ammonia in the nitrogen fixation process [35]. Finally, *Nitrospira*, a bacterium related to the entire nitrification process, was found more frequently in CF systems [36].

### 3.3. Diversity Analyses and Multiple Factor Analysis (MFA)

**Figure 4:**
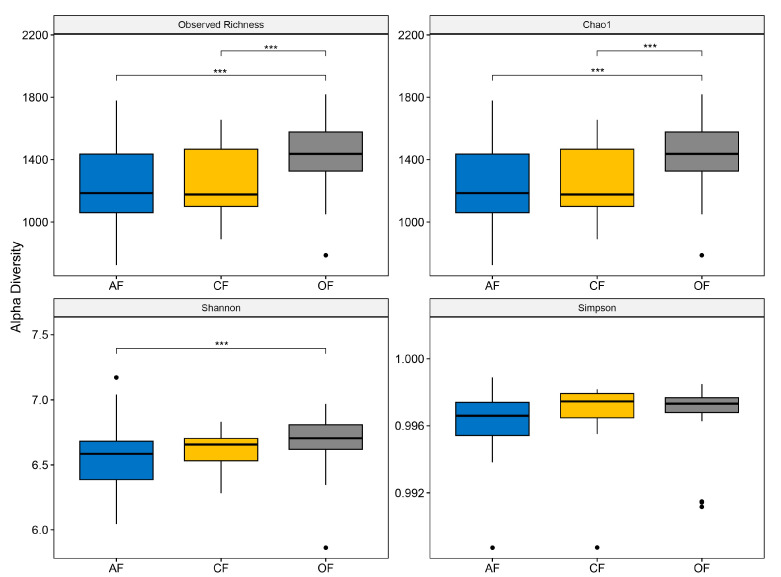
Alpha diversity indices of soil microbial communities in the three production systems. *** *p* < 0.05.

The values in the alpha diversity indices were similar across the three production systems (Figure 4). Accordingly, AF (1529 Chao1 richness index; 6.711 Shannon’s index; 0.997 Simpson dominance index) showed the highest species richness, followed by CF (1348; 6.670; 0.997), and finally, AF (1319; 6.612; 0.996) (Figure 4 and Table S3). Considering these results, all three soil types show moderate disturbance, inferred to be from tillage systems. On the other hand, the bacterial richness indicates that there is low competition between populations [37], which can be explained by the diversity of plants in agroecological systems and mineral fertilization in organic and conventional systems [38,39].

**Figure 5:**
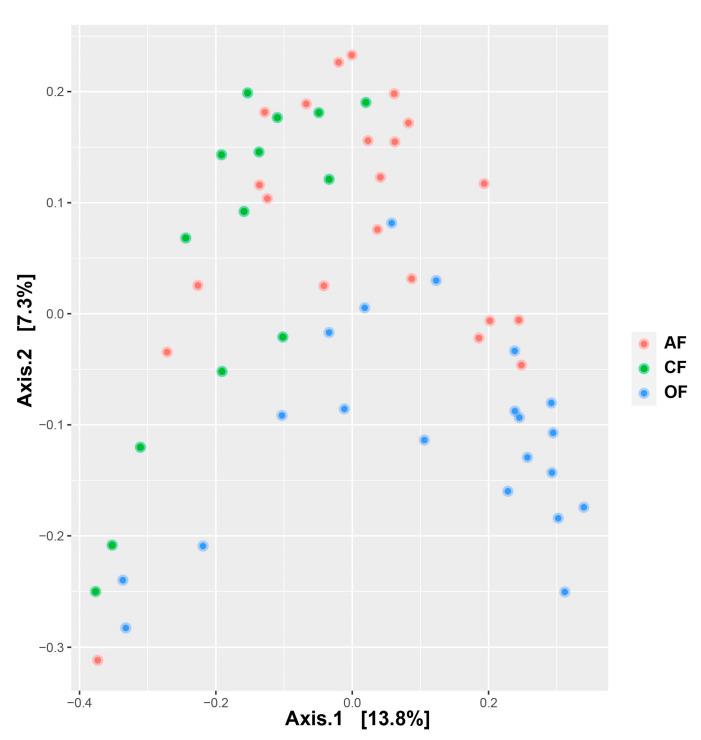
Principal coordinate analysis (PCoA) using the Bray–Curtis distance on relative abundance for 57 samples across three production systems.

The β diversity was evaluated with the Bray–Curtis distance, which refers to the total difference in the abundance of species between two sites [40]. In the PCoA analysis (Figure 5) with all sampled sites (57), the first axis accounted for 13.9% of the variance in community composition and discriminates between agricultural production systems, but with a low coefficient of determination (PERMANOVA, F = 3.06; R^2^ = 0.10; *p* = 0.001), while the second axis explained 7.3% of the variance. The bacterial community composition differed between agricultural production systems in terms of relative abundance (Figure S1).

**Figure 6:**
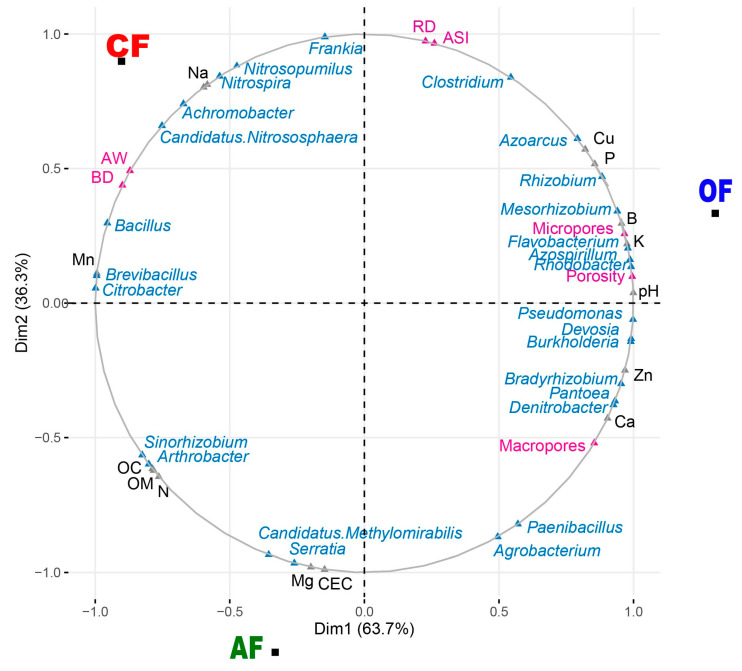
Multiple factor analysis (MFA) plot for biological bacteria genera (*Achromobacter*, *Agrobacterium*, *Arthrobacter*, *Azoarcus*, *Azospirillum*, *Bacillus*, *Bradyrhizobium*, *Brevibacillus*, *Burkholderia*, Candidatus *Methylomirabilis*, Candidatus *Nitrososphaera*, *Citrobacter*, *Clostridium*, *Denitrobacter*, *Devosia*, *Flavobacterium*, *Frankia*, *Mesorhizobium*, *Nitrosopumilus*, *Nitrospira*, *Paenibacillus*, *Pseudomonas*, *Rhizobium*, *Rhodobacter*, *Serratia*, *Sinorhizobium*, and *Pantoea*; blue) and chemical (N, Na, Mg, Mn, Ca, Cu, Zn, P, B, K, Fe, pH, CEC, CO, and MO; gray) and physical (macropores, micropores, porosity, stability index, real density, apparent density, and available water; pink) variables for the three production systems.

Using the data obtained in the physicochemical analyses of the soil and the bacterial genera that play an important role in the soil (Table S2), a multiple factorial analysis was carried out to simultaneously analyze all the variables (chemical, physical, and bacteria) and to attribute the contribution of each of these to the different systems (agroecological, organic, and conventional). In Figure 6, dimension one explains 63.7% of the total variance of the data, separating OF from the CF and AF systems, while dimension two with a variance of 36.3% separates the OF and CF systems from the AF. Regarding the quantitative variables, the variables with the greatest contribution in dimension one in the OF system are (i) physical: micropores (7.19) and porosity (7.62); (ii) chemical: pH (3.38), potassium (3.22), boron (3.09), phosphorus (2.48), and copper (2.28); and (iii) bacteria: *Pseudomonas* (1.92), *Devosia* (1.89), *Burkholderia* (1.89), *Rhodobacter* (1.89), *Azospirillum* (1.88), *Flavobacterium* (1.85), *Bradyrhizobium* (1.75), *Mesorhizobium* (1.70), and *Rhizobium* (1.50). For dimension two in the OF system, no significant values were found. In the CF system, the greatest contributions for dimension one are (i) physical: apparent density (6.22) and available water (5.83); (ii) chemicals: manganese (3.35); and (iii) bacteria: *Frankia* (3.31) and *Bacillus* (1.76), while the greatest contributions made to dimension two by variables are (i) chemicals: iron (1.21) and sodium (1.15) and (ii) bacteria: *Nitrosopumillus* (2.63), *Nitrospira* (2.41), *Achromobacter* (1.85), and Candidatus *Nitrososphaera* (1.47). Finally, for AF systems, the contributions for dimension one correspond to (i) chemicals: organic matter (2.10), organic carbon (2.07), and nitrogen (1.98) and (ii) bacteria: *Arthrobacter* (1.31) and *Sinorhizobium* (1.24). The contributions in dimension two were (i) chemicals: CEC (5.82) and magnesium (5.72) and (ii) bacteria: Candidatus *Methylomirabilis* (2.96) and *Serratia* (3.16).

## 4. Discussion

Chemical analyses of the soils showed that organic matter, nitrogen, and organic carbon were higher in the agroecological systems. This is due to the abundant presence of weeds and crop residues, while herbicides or the controlled presence of weeds are used in the other systems. The values of Ca, K, and Na were high in the conventional systems due to the use of chemical fertilizers that provide these minerals to the soil, while Mg was higher in the agroecological systems. In general, these minerals are related to soil fertility and especially to nutrient storage and cation exchange capacity (CEC) (i.e., the number of sites available to store cations needed in the nutritional processes of the plant) [30]. The CEC is directly related to the amount of organic matter [41] and type of clay present in the soil, as well as to the organic matter content [42]. Phosphorus showed high levels in agroecological and organic systems, while in conventional systems they were excessive. Microelements such as Fe (82.6), Cu (4.71), and Mn (11.2) registered high values in organic systems, likely due to the application of minerals that help in the production of fruits and the synthesis and formation of chlorophyll [42].

In evaluating the physical parameters of the soils, the agroecological systems were found to be less susceptible to soil erosion, as they have a greater source of organic matter and the presence of accompanying plants that, together with clay and silt particles, form macro aggregates, giving greater stability to the soils [42]. In the organic and conventional systems, cleaner soils were observed, indicating the loss of fine soil particles by runoff and presenting a greater risk of erosion.

We found that the most abundant phylum in all three systems was proteobacteria, with values of 28.1%, 30.4%, and 27% for AF, CF, and OF systems, respectively. This phylum is often abundant in the rhizospheres of some plants, and it plays a key role in carbon cycles and nitrogen and sulfur fixation [43]. The second most abundant phylum was Acidobacteria, with 21.5%, 21.4%, and 24.1% in AF, OF, and CF systems, respectively. These bacteria are common in mainly acidic soils [44]. Other phyla present that fulfil an important function within the soil are related to the practices developed in each of these systems, such as the use of chemical fertilizers rich in nitrogen and phosphorus (OF) and the use of herbicides and pesticides (CF). AF systems have a great variety and abundance of these ASVs, mainly due to tillage management, accompanying plants, and the use of some biofertilizers.

In their study “Structure, composition and metagenomic profile of soil for agricultural and farm use” [45], Carbonetto, Rascovan, Álvarez, Mentaberry, and Vázquez found that the most abundant phyla in uncultivated soils were Verrucomicrobia, Plactomycetes, Actinobacteria, and Chloroflexi, while Gemmatimonadetes, Nitrospirae, and WS3 were the most abundant in cultivated soils. Fierer, Bradford, and Jackson [46] suggest that a high number of oligotrophic prokaryotes are found in soils with high amounts of recalcitrant organic matter, while [47] found that Proteobacteria, Firmicutes, Acidobacteria, and Actinobacteria were associated with different stages of the nitrogen cycle. On the other hand, in a study assessing the impact of crops on soil bacterial and fungal diversity in tropical grasslands, Lienhard et al. [37] found that tillage performed on crops significantly decreased the relative abundance of Actinobacteria, Acidobacteria, and Delta-proteobacteria, while the presence of Firmicutes, Gamma-proteobacteria, and Chytridiomycota increased. Finally, the main groups of plant-growth-promoting bacteria (PGPB) were found to include Cyanobacteria, Actinobacteria, Bacteroidetes, Firmicutes, and Proteobacteria [48].

The alpha diversity index values obtained were similar across the three production systems. Considering these results, the soils are inferred to be under moderate disturbance from tillage [37]. Plant diversity in the agroecological systems and mineral fertilization in the organic and conventional systems may also have contributed to the high bacterial diversity found [38,39]. At the beta diversity level, it was observed that the three systems have shared ASVs, which corroborates that the bacterial diversity is similar between the systems. This may be explained by their close geographic proximity and relatively low levels of disturbance. With similar physical and chemical characteristics of soils, no groupings are observed between production systems.

Finally, the multifactorial analyses showed a relation between the organic systems with bacteria that fix nitrogen. These include *Burkholderia*, a free-living nitrogen-fixing bacterium [49]; *Devosia*, bacterial symbionts that form nitrogen-fixing root nodules in the legumes [50]; *Rhizobium*, symbiotic nitrogen fixers and root nodule-formers [51]; *Flavobacterium*, that have potential as nitrogen-fixing microorganisms [52]; *Denitrobacter*, which are microorganisms that consume nitrites and produce nitrates [44]; *Bradyrhizobium*, which fix nitrogen [53]; *Azoarcus*, non-symbiotic nitrogen-fixing bacteria whose attachment is by free-living, associative, or endophytic diazotrophs [51]; and *Rhodobacter*, a red bacterium that can fix nitrogen [33]. These are likely present due to the application of minor elements to produce fruits and some nitrogenous elements within urea. Non-symbiotic nitrogen (N2) fixation by diazotrophic bacteria has been found to be a potential source of biological input of N in pastures, while some weed species provide adequate microsites with the required carbon supply for N2 fixation by diazotrophs [54]. In our study, the greatest contributions of these species in the organic sites were found in the Guadalajara farm, which through its citrus cultivation manages cut pastures for feeding cattle, sheep, and goats.

Agroecological systems are linked to an abundant production of organic matter, which through the decomposition process increase organic carbon and nitrogen and the cation exchange capacity. The genera grouped in this system were Candidatus *Methylomirabilis* bacterium of the phylum NC10, which combine methane oxidation with denitrification through a recently discovered intra-aerobic pathway [55]; *Serratia*, which are characterized by being phosphate solubilizers [56]; and free-living nitrogen-fixing *Sinorhizobium* [57].

Finally, conventional systems harbored soils with signs of chemical fertilizer application, which is linked to the presence of bacteria that solubilize phosphates and fix nitrogen, such as *Bacillus* growth promoters, free-living nitrogen fixers, and phosphate solubilizers [33,57]; ammonia-oxidizing Candidatus *Nitrososphaera* archaeas in the order Nitrososphaerales [35,58]; *Nitrospira,* chemolithoautotrophic microorganisms that oxidize ammonia or nitrite to nitrate [36]; *Frankia*, which are filamentous nitrogen-fixing bacteria that live in symbiosis with plants [59]; and *Nitrosopumilus*, which oxidize ammonia to nitrite [60]. In these systems, tomato crops (*Solanum lycopersicum* L.) were predominant. About 50% of the rhizosphere of tomato plants have been found to be colonized by bacteria of the phylum Proteobacteria and Bacteroidetes, with bacteria of the genus *Flavobacterium* (Bacteroidetes) being the dominant colonizers, which play a role in the mineralization of various types of organic matter [61]. However, it is not yet known whether their presence is linked to management practices.

## 5. Conclusions

Physicochemical and metabarcoding analyses revealed that bacterial diversity is related to the management of each agricultural system; however, no differences were found in the relative abundances between each system. This reflects that the developed practices are not very aggressive. The differences between systems are as follows: fertilizers rich in nitrogen and phosphorus are used in organic systems; pesticides and chemical fertilizers are used in conventional systems; and tillage management, accompanying plants, and some biofertilizers are used in agroecological systems. A multiple factorial analysis (MFA) separated the production systems, observing the contributions of each variable for each system. The physical, chemical, and bacterial variables grouped the systems according to each management type, allowing them to be differentiated and the interactions between each of them to be assessed.

## Figures and Tables

**Table 1:** Metadata of soil samples collected at organic, agroecological, and conventional farms.

Farm	Location	Altitude (MASL)	Area (ha)	Farm Type	Sampling Points
Guadalajara	Buitrera	1303–1307	3.6	Organic (OF_1)	6
La Esmeralda	Arenillo	1695–1830	6.8	Organic (OF_2)	8
Esmeralda	El Mesón	1580–1596	1.0	Organic (OF_3)	7
El Mesón	El Mesón	1607–1662	3.4	Agroecological (AF_1)	10
El Sendero	El Mesón	1644–1722	3.2	Agroecological (AF_2)	12
El Descanso	Arenillo	1679–1686	0.15	Conventional (CF_1)	4
El Paraíso No.1	El Mesón	1576–1584	0.13	Conventional (CF_2)	5
El Paraíso No.2	El Mesón	1630–1672	0.39	Conventional (CF_3)	5

**Table 2:** Physicochemical analyses results at conventional, agroecological, and organic farms.

Physicochemical Variables	Agroecological Farm (AF)	Organic Farm (OF)	Conventional Farm (CF)
pH (1:1; p/v)	6.12	6.08	6.6
Organic Carbon—OC (%)	3.55	3.24	2.78
Organic matter—OM (%)	6.12	5.59	4.78
Nitrogen—N (%)	0.31	0.28	0.24
Calcium—Ca (meq/100 g)	25.39	22.23	27.04
Magnesium—Mg (meq/100 g)	13.08	8.26	8.6
Potassium—K (meq/100 g)	0.46	0.42	1.33
Sodium—Na (meq/100 g)	0.07	0.09	0.13
Cation Exchange Capacity—CEC (meq/100g)	39.47	38.03	38.21
Phosphorus—P (ppm)	43.56	54.9	82.97
Copper—Cu (mg/kg)	3.65	4.71	3.71
Zinc—Zn (mg/kg)	4.36	2.26	6.54
Manganese—Mn (mg/kg)	8.38	11.25	3.43
Iron—Fe (mg/kg)	30.1	82.59	38.22
Boron—B (mg/kg)	0.04	0.08	0.92
Bulk density—BD (g m^3^)	0.9	0.89	0.92
Real density—RD (g cm^3^)	2.2	2.23	2.23
Total porosity (g cm^3^)	59.34	60.35	59.13
Macroporosity (%)	26.08	26.78	23.95
Microporosity (%)	33.26	33.57	33.26
Aggregate stability index—ASI	0.19	0.44	0.45
Available water (mm)	26.36	26.11	27.01

## Data Availability

Not applicable.

## Supplementary Materials

The following supporting information can be downloaded at: https://www.mdpi.com/article/10.3390/biology12050701/s1, Table S1: Sampling sites and accession numbers. Table S2: Taxonomic assignment of the bacterial communities. Table S3: Contribution values for the multifactorial analysis and their significance values. Figure S1. Log-transformed ratio of the relative abundance of bacterial genera between agrosystems estimated by DESeq2.
