# Supplementary figures and images for: Soil Bacterial Communities from Three Agricultural Production Systems in Rural Landscapes of Palmira, Colombia
Source: Biology (Basel). 2023 May 11;12(5):701. doi: 10.3390/biology12050701 (PMC10215842; doi:10.3390/biology12050701)

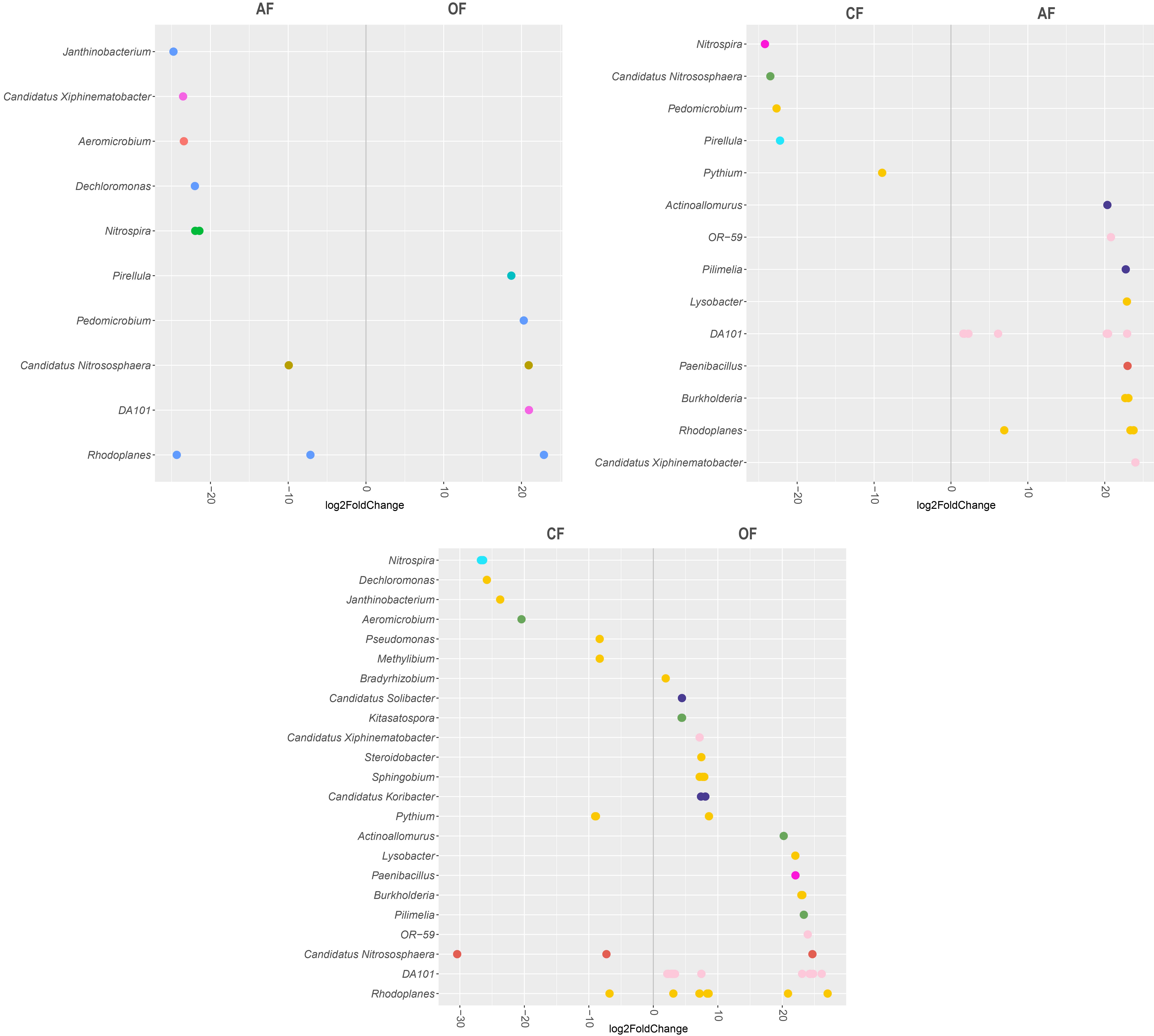

Supplement: Supplementary file 1 [file biology-12-00701-s001.zip › Figure_S1.jpg]
